# Coding and noncoding variants in *EBF3* are involved in HADDS and simplex autism

**DOI:** 10.1186/s40246-021-00342-3

**Published:** 2021-07-13

**Authors:** Evin M. Padhi, Tristan J. Hayeck, Zhang Cheng, Sumantra Chatterjee, Brandon J. Mannion, Marta Byrska-Bishop, Marjolaine Willems, Lucile Pinson, Sylvia Redon, Caroline Benech, Kevin Uguen, Séverine Audebert-Bellanger, Cédric Le Marechal, Claude Férec, Stephanie Efthymiou, Fatima Rahman, Shazia Maqbool, Reza Maroofian, Henry Houlden, Rajeeva Musunuri, Giuseppe Narzisi, Avinash Abhyankar, Riana D. Hunter, Jennifer Akiyama, Lauren E. Fries, Jeffrey K. Ng, Elvisa Mehinovic, Nick Stong, Andrew S. Allen, Diane E. Dickel, Raphael A. Bernier, David U. Gorkin, Len A. Pennacchio, Michael C. Zody, Tychele N. Turner

**Affiliations:** 1 Department of Genetics, Washington University School of Medicine, 4523 Clayton Avenue, Campus Box 8232, St. Louis, MO 63110 USA; 2 Department of Pathology and Laboratory Medicine, Perelman School of Medicine, University of Pennsylvania, Philadelphia, PA 19104 USA; 3 Department of Pathology and Laboratory Medicine, Children’s Hospital of Philadelphia, Philadelphia, PA 19104 USA; 4 Center for Epigenomics, University of California San Diego School of Medicine, 9500 Gilman Drive, La Jolla, CA 92093 USA; 5 Center for Human Genetics and Genomics, NYU School of Medicine, New York, NY 10016 USA; 6 Environmental Genomics and Systems Biology Division, Lawrence Berkeley National Laboratory, Berkeley, CA 94720 USA; 7 New York Genome Center, New York, NY 10013 USA; 8 University of Montpellier, département de Génétique, maladies rares médecine personnalisée, U 1298, CHU Montpellier, University of Montpellier, Montpellier, France; 9 CHU Brest, Inserm, Univ Brest, EFS,UMR 1078, GGB, F-29200 Brest, France; 10 Service de Génétique Médicale, CHRU de Brest, Brest, France; 11 Department of Neuromuscular Disorders, UCL Institute of Neurology, Queen Square, London, WC1N 3BG UK; 12 Development and Behavioral Pediatrics Department, Institute of Child Health and Children Hospital, Lahore, Pakistan; 13 Institute for Genomic Medicine, Columbia University, New York, NY 10027 USA; 14 Center for Statistical Genetics and Genomics, Duke University, Durham, NC 27708 USA; 15 Division of Integrative Genomics, Duke University, Durham, NC 27708 USA; 16 Department of Biostatistics and Bioinformatics, Duke University, Durham, NC 27708 USA; 17 Department of Psychiatry and Behavioral Sciences, University of Washington, Seattle, WA 98195 USA; 18 Department of Biology, Emory University, Atlanta, GA 30322 USA; 19 U.S. Department of Energy Joint Genome Institute, Walnut Creek, CA 94598 USA

**Keywords:** Autism, Neurodevelopmental disorder, Enhancer, Gene regulatory network, *EBF3*, hs737, Genome, Variant, De novo

## Abstract

**Background:**

Previous research in autism and other neurodevelopmental disorders (NDDs) has indicated an important contribution of protein-coding (coding) de novo variants (DNVs) within specific genes. The role of de novo noncoding variation has been observable as a general increase in genetic burden but has yet to be resolved to individual functional elements. In this study, we assessed whole-genome sequencing data in 2671 families with autism (discovery cohort of 516 families, replication cohort of 2155 families). We focused on DNVs in enhancers with characterized in vivo activity in the brain and identified an excess of DNVs in an enhancer named hs737.

**Results:**

We adapted the fitDNM statistical model to work in noncoding regions and tested enhancers for excess of DNVs in families with autism. We found only one enhancer (hs737) with nominal significance in the discovery (p = 0.0172), replication (p = 2.5 × 10^−3^), and combined dataset (p = 1.1 × 10^−4^). Each individual with a DNV in hs737 had shared phenotypes including being male, intact cognitive function, and hypotonia or motor delay. Our in vitro assessment of the DNVs showed they all reduce enhancer activity in a neuronal cell line. By epigenomic analyses, we found that hs737 is brain-specific and targets the transcription factor gene *EBF3* in human fetal brain. *EBF3* is genome-wide significant for coding DNVs in NDDs (missense p = 8.12 × 10^−35^, loss-of-function p = 2.26 × 10^−13^) and is widely expressed in the body. Through characterization of promoters bound by EBF3 in neuronal cells, we saw enrichment for binding to NDD genes (p = 7.43 × 10^−6^, OR = 1.87) involved in gene regulation. Individuals with coding DNVs have greater phenotypic severity (hypotonia, ataxia, and delayed development syndrome [HADDS]) in comparison to individuals with noncoding DNVs that have autism and hypotonia.

**Conclusions:**

In this study, we identify DNVs in the hs737 enhancer in individuals with autism. Through multiple approaches, we find hs737 targets the gene *EBF3* that is genome-wide significant in NDDs. By assessment of noncoding variation and the genes they affect, we are beginning to understand their impact on gene regulatory networks in NDDs.

**Supplementary Information:**

The online version contains supplementary material available at 10.1186/s40246-021-00342-3.

## Background

Large-scale whole-genome sequencing (WGS) is becoming instrumental in assessing the contribution of protein-coding (coding), but more importantly noncoding variants in complex diseases [1]. Unlike coding exons, the boundaries of noncoding regions are not well defined and hence different types of annotations including but not limited to evolutionary conservation [2], sequence constraint [3], and epigenetic marks [4] are useful guides. Though genome-wide association studies (GWAS) have identified multiple common noncoding variants associated with human disorders [5–7], WGS has now provided access to rare de novo variants (DNVs) which are difficult to associate with phenotype without using aggregation methods [8–11]. These aggregation methods have been successfully used for rare coding variants [12–16], but have been challenging for noncoding regions because of the lack of clearly defined, discrete genomic boundaries and sequence-based models of variant effect.

Autism is a complex neurodevelopmental disorder with a heritability of ~ 80% [17]. Large copy number variants [18–21] and coding DNVs contribute to ~ 30% of cases with higher enrichment in females with autism and those with intellectual disability [12–16, 22, 23]. Recently, we and others have identified an overall enrichment of de novo [8–11, 24] or paternally inherited variants [25] within the regulatory sequence of individuals with autism. However, these studies have mostly assessed this aggregation of genetic burden across a large panel of pooled regulatory elements. To begin to parse out the underlying biology of autism DNVs in individual regulatory regions, we turned to VISTA, which is a database of functionally characterized developmental enhancers [26–28]. These enhancers were identified based on multiple strategies including sequence conservation and epigenetic signatures. Each enhancer has been tested in transgenic mouse assays providing information on the spatial-temporal dynamics of their activity during mammalian development. We adapted the fitDNM model [29] that was previously used to test for excess DNV load in coding regions so that it would work in noncoding regions. We then applied our updated version of the fitDNM model to VISTA enhancers with known ability to drive expression in the embryonic brain. Application of this test in 2671 families with autism (n = 9831 individuals) revealed one VISTA enhancer (named hs737) with nominal significance for excess of DNVs in autism in our discovery cohort (516 families), replication cohort (2155 families), and the combined dataset. We extensively tested enhancer hs737 in follow-up genomic, epigenomic, phenotypic, in silico, and in vitro analyses. Our analysis revealed this enhancer targets the transcription factor gene *EBF3* which is enriched for coding DNVs in the hypotonia, ataxia, and delayed development syndrome (HADDS). We also identified new patients with coding variants in *EBF3* and performed extensive phenotypic analysis. Combining this with phenotype data we collected for individuals with the hs737 enhancer DNVs, we found marked increases in phenotypic severity in individuals with coding than noncoding variants. This work provides critical insights into coding and noncoding DNVs at *EBF3* and more generally in neurodevelopmental disorders.

## Results

### Statistical assessment of DNVs in VISTA elements

To assess for DNVs in individuals with autism, we aggregated DNV data from a WGS study of 2671 families with autism [30]. To test the enrichment of DNVs in noncoding regions, we modified the existing fitDNM [29]. In particular, we focused on 544 VISTA human noncoding enhancers (Supplemental Table S1, Supplemental Table S2) previously shown to have enhancer activity in the brain using a lacZ transgenic assay at embryonic day 11.5 in mice [27]. We assessed these same enhancers in our previous paper (Turner et al. [8]) studying 516 families (discovery cohort) and we wanted to test whether any of these enhancers replicated in a new set of 2155 families (replication cohort). Of these enhancers, we identified one (hs737, see Table 1) reaching nominal significance in both cohorts (discovery p = 0.0172, replication p = 2.5 × 10^−3^) and in the combined dataset (p = 1.1 × 10^−4^). The hs737 enhancer drives expression in the midbrain and hindbrain at embryonic day 11.5 (E11.5) [8].

**Table 1:**
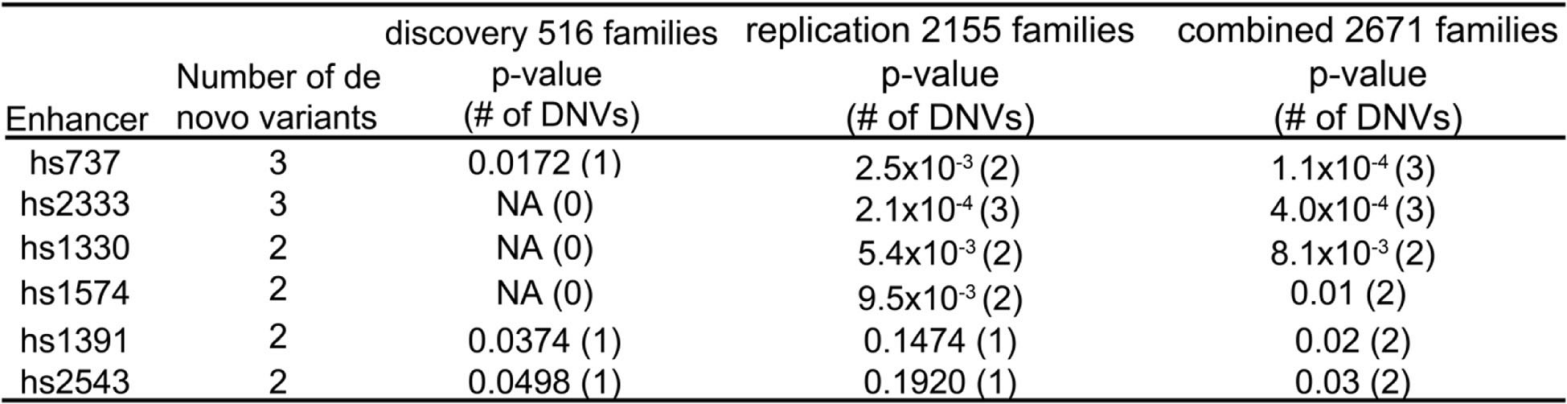
VISTA enhancers with an excess of de novo mutation based on fitDNM analysis

In each individual with a DNV in hs737, we also assessed the rest of the genome for other potentially relevant genomic variants that could be contributing to autism in each of the individuals (Supplemental Table S3, S4). Individual 13396.p1 had no other large de novo CNVs or coding de novo SNVs/indels. Individual 12975.p1 had three total de novo missense variants with one in each of the following genes *CHD6*, *FAM129B*, and *KCNC1* and also had a 1.6-Mbp de novo deletion at 11q24.1 containing the following genes *BLID*, *BSX*, *C11orf63*, *CRTAM*, *SORL1*, and *UBASH3B*. Individual 11257.p1 had two de novo missense variants in each of the following genes *RUVBL1* and *VKORC1L1*. We scored each of the variants using a clinical variant scoring program (https://franklin.genoox.com/clinical-db/home) and all of the variants were classified as variants of uncertain significance. We checked each individual’s polygenic risk score [31] for autism spectrum disorder, schizophrenia, and educational attainment and find no significant contribution for any of the three individuals (Supplementary Figure 1).

### In vitro assessment of hs737 DNVs

**Fig. 1:**
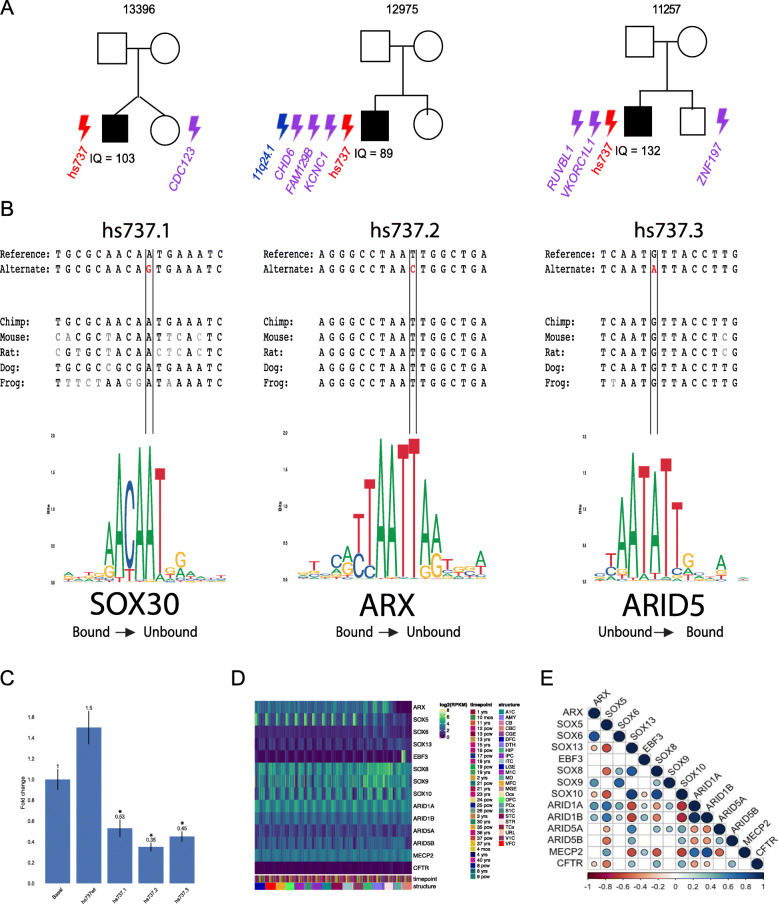
Characterization of DNVs in hs737. **A** Pedigrees of families with de novo variants in hs737. Lightning symbols indicate de novo variants with red = regulatory, purple = missense, and blue = deletion. Family identifiers are shown above the pedigree and the full-scale IQ is shown below each proband. **B** Sequence analysis of each of the three hs737 de novo mutations, identified in individuals with autism, including transcription factor binding site analysis results. **C** Results of luciferase assays in neuroblastoma (Neuro2a) cell lines with rs2435357 (RET+3) as a positive control for enhancer activity, promoter only (Basal), the wild type sequence of hs737 (hs737wt), and each of the three DNVs identified in individuals with autism. Error bars represent standard error (SE). **D** log2 normalized expression of genes from the transcription factor binding site analysis in the brain throughout development and adulthood. **E** Correlogram of candidate genes and *EBF3* after performing regression, with positive control *MECP2* and negative control *CFTR*

In order to quantify the in vitro transcriptional effects of hs737 DNVs, we transfected the neuronal cell line Neuro2a with a reporter construct that had both the non-risk and risk allele of the enhancer individually cloned upstream of a luciferase gene and a minimal promoter [32]. As a control, we examined the expression of the known RET+3 enhancer, shown to be functionally active in this cell line [32]. Our data shows that all DNVs in hs737 led to a significant reduction in reporter gene expression when compared to their respective non-risk allele and the promoter-only construct and the control enhancer (RET+3) had high transcriptional activity (Fig. 1C). Thus, these DNVs in hs737 can individually affect the transcription of their cognate gene.

### In silico transcription factor binding assessment

To better understand functional consequences of the hs737 variants we observed, we analyzed the reference and variant enhancer sequences using QBiC [33, 34] (http://qbic.gcb.duke.edu), a program designed to predict the impact of noncoding mutations on transcription factor binding sites. We found that the DNVs identified in individuals with autism were each predicted to impact transcription factor binding (Fig. 1B). Here we report the most significant hits, realizing that it could be any of the transcription factors in the family causing the functional impact on expression dynamics. All three variants are predicted to impact transcription factor binding via a transition mutation at a highly preferential base that is also highly conserved to frog (Fig. 1B). Each mutation occurs at a location within the position weight matrix that is predicted to completely change the binding status of the transcription factors (Fig. 1B). The first two variants are predicted to each respectively cause SOX30 and ARX to go from the bound state to unbound (SOX30 p = 2.48 × 10^−189^, z-score = −29.35; ARX p < 2.2 × 10^−16^, z-score = −40.27) while the third variant is predicted to create a new binding site for ARID5 (p < 2.2 × 10^−16^, z-score = 31.87). Mouse RNAseq at day E11.5 (see the “Methods” section) provides further support for these transcription factors being impactful as ARX and 12 members of the SOX family are in the 90th percentile and the ARID family members are in the 80th percentile of all genes that are expressed in the brain (Supplemental Table S6). We specifically assessed RNAseq data at E11.5 since that is the timepoint at which lacZ reporter expression was tested and observed in the VISTA enhancer database for the hs737 enhancer (http://enhancer.lbl.gov/cgi-bin/imagedb3.pl?form=presentation&show=1&experiment_id=737&organism_id=1).

Next, we turned to human RNAseq data from Brainspan (http://brainspan.org/rnaseq/search/index.html), which contains expression profiles from various timepoints ranging from early in development (8 post-conception weeks [pcw]) to adulthood (40 years) and examines many regions of the brain. We wanted to determine what transcription factor may be binding to the enhancer. As we describe below, the target gene of hs737 is *EBF3*. We hypothesized that the transcription factor that is binding may be correlated in expression with *EBF3*. We find that *EBF3* is expressed throughout the brain prenatally and is most highly expressed through the hindbrain in the cerebellum and cerebellar cortex (Fig. 1D). We find further evidence for the transcription factors implicated by the in silico analysis. *EBF3* expression is significantly correlated with *SOX10* and the *ARID1* family after performing a linear regression for age and brain region (Fig. 1E).

### Dosage sensitivity of hs737 in the human genome

**Fig. 2:**
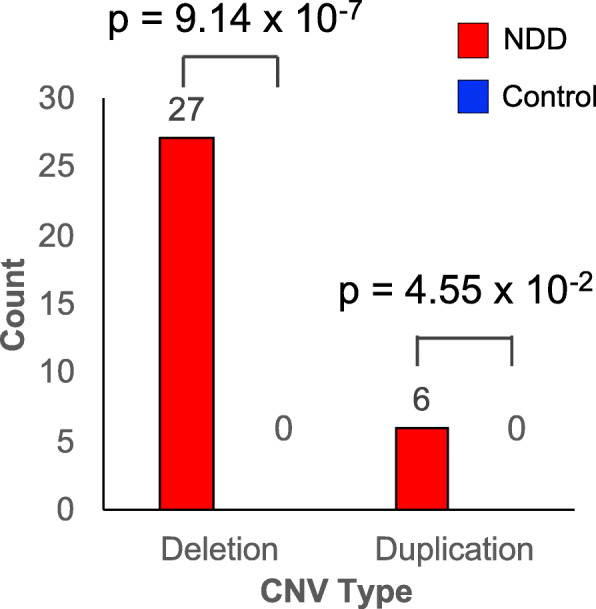
Copy number variation over hs737. Displays the counts for both deletions and duplications over hs737 in individuals with neurodevelopmental disorders and controls

To further assess the impact of enhancer hs737 on NDDs, we measured the effect of its dosage on the NDD phenotypes. We hypothesize that if heterozygous point mutations in hs737 alter phenotype strongly, the enhancer would be dosage sensitive and show copy number variation (CNV) only in individuals with an NDD. To test this, we first applied two approaches to assess CNVs in hs737. We assessed the morbidity map [35, 36] database containing 29,085 individuals with neurodevelopmental disorders (NDDs) and 19,584 controls. In particular, we looked at the window analysis in Coe et al. [35] across the genome to identify the window containing hs737. This enhancer resided in a genomic window with an excess of deletions (case counts = 27, control counts = 0, p = 9.14 × 10^−7^) and duplications (case counts = 6, control counts = 0, p = 4.55 × 10^−2^) in individuals with NDDs. None of the 19,584 control individuals contained a CNV in this enhancer (Fig. 2). On average, these CNVs containing hs737 were 10,570,885 ± 5,789,023 bp long and overlapped 70 genes (Supplemental Table S7). The smallest CNV was 161 kbp and was in completely noncoding space.

We also applied a tool to determine copy number (with paralog-specific sensitivity) in 1-kbp windows across the whole genome [37] to our WGS data and identified only one CNV in this enhancer. It was a deletion and occurred in proband 14091.p1 and upon further inspection was found to be part of a larger known deletion (hg38: chr10:126450330–133655780) [38]. To determine the frequency of deletion/duplication in this enhancer in individuals without autism, we also ran this copy number approach on the newly generated 3202 individuals (high-coverage WGS) from the 1000 genomes project [39]. Combining our WGS parental data, 1000 genomes project data, and morbidity map, there are no deletions or duplications in this enhancer in 28,128 non-NDD individuals (56,256 alleles). We also surveyed the gnomAD [40] database (v2.1) and observed no CNVs in the 10,847 individuals contained there (n = 21,694 alleles). To avoid possible double counting between gnomAD and other datasets, we do not present the aggregate data. Taken together, these results suggest that in addition to point mutations, CNVs involving hs737 may play a significant role in NDDs.

### Epigenetic characteristics of hs737

Enhancers have well-characterized epigenetic signatures that are predictive of their activity in specific biological contexts. Thus, to examine the activity of hs737 in its native genomic context, we took advantage of available epigenomic datasets from relevant human samples. We found that hs737 has several hallmarks of neuronal enhancer activity in humans including H3K27ac enrichment in fetal brain tissue [41], DNaseI hypersensitive sites (DHS) indicative of chromatin accessibility in CNS tissues [8, 24], and conserved transcription factor binding sites (TFBS) [8] (HMR conserved transcription factor binding sites track in the UCSC Genome Browser [42]) (Supplemental Figure S2).

**Fig. 3:**
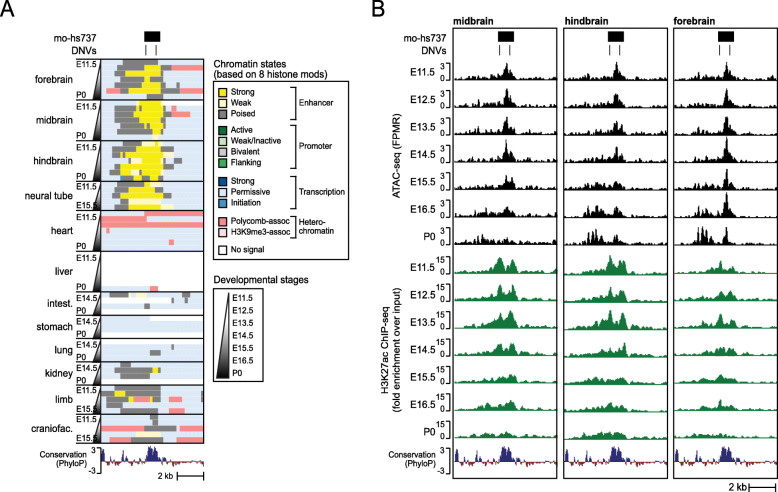
hs737 is a prenatal, brain-specific enhancer. **A** Genome browser view (chr7:136,079,964–136,087,591; mm10) of chromatin states in mouse from [43] called by chromHMM [44] based on eight histone modifications: H3K4me1, H3K4me2, H3K4me3, H3K27ac, H3K27me3, H3K36me3, H3K9me3, and H3K9ac. **B** Genome browser view (chr7:136,079,964–136,087,591; mm10) of ATAC-seq and H3K27ac ChIP-seq signal in midbrain, hindbrain, and forebrain at multiple developmental mouse stages from E11.5 to the day of birth (P0)

Hs737 was assessed for reporter activity in mouse embryos at E11.5 in the VISTA database. To further examine the activity of this element during development, we analyzed the orthologous region in the mouse genome (mouse ortholog of hs737, or mo-hs737) using epigenomic data from a mouse embryonic developmental time series recently published [43–45]. Consistent with the reporter expression pattern of hs737, we found that mo-hs737 has the chromatin signature of an active enhancer in the midbrain and hindbrain at E11.5, but not in the non-neuronal tissues assayed (Fig. 3A).

Strikingly, we found that characteristics of enhancer activity at mo-hs737 such as H3K27ac and chromatin accessibility (as measured by ATAC-seq) reach their height in brain tissue in mid to late gestation and decline at birth (Fig. 3B). This suggests that hs737/mo-hs737 may exert its regulatory influence specifically during embryonic development, which could explain its involvement in NDDs, and may point to developmental stages and model systems that are most appropriate for future studies of this element.

### Gene target of hs737

**Fig. 4:**
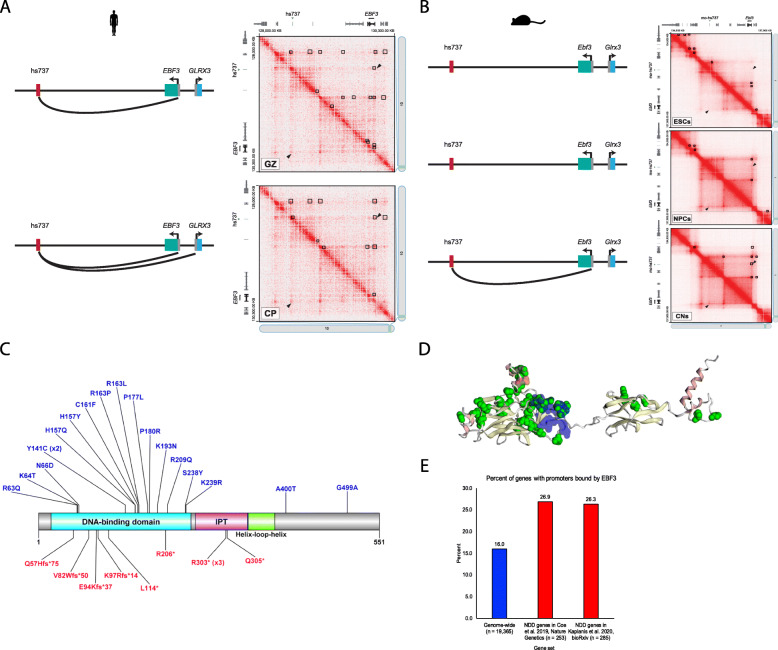
*EBF3* is the gene target of hs737. **A** Schematic of hs737 and target genes. Gray boxes represent promoters and colored boxes represent gene bodies and red box represents hs737. Hi-C contact map generated using data from Won et al. [49] visualized with Juicebox [48] at 25 kbp. Heatmaps are symmetrical across the diagonal, except that HiCCUPS loop calls are shown as black boxes in the upper right half of each heatmap. **B** Hi-C contact maps from Bonev et al. [47] visualized with Juicebox [48] at 5-kbp resolution. **C** EBF3 protein diagram (plotted using the DOG protein plotter [67]) with DNVs identified in NDDs. Shown in blue are the missense variants and in red are the loss-of-function variants. **D** 3D model (plotted using the MuPIT program [68, 69]) of the EBF3 protein with DNVs identified in individuals with NDDs shown in green. **E** Genes with promoters bound by EBF3 based on ChIP sequencing in SK-N-SH cells. Enrichment is seen for the promoters of known NDD genesets

To determine the potential gene target of the hs737 enhancer, we first looked at all of the genes residing within the same topologically associating domain (TAD) (hg38, chr10:128151746–130191746) [46]. We focused on these genes since they are the most likely to be the targets of this enhancer. The genes included *C10orf143*, *CTAGE7P*, *EBF3*, *GLRX3*, *LINC01163*, *LOC728327*, *MGMT*, and *MIR4297*; the only gene in this region that is constrained in the human population with a gnomAD [3] o/e value for loss-of-function SNVs of 0.03 is *EBF3*. To examine physical interactions between hs737/mo-hs737 and genes in this region, we analyzed two separate high-resolution Hi-C datasets examining the 3D architecture of the genome throughout mouse neuronal differentiation and human fetal corticogenesis. The mouse dataset was generated in a differentiation course from mouse embryonic stem cells (ESCs) to neural progenitor cells (NPCs) and then to cortical-like neurons (CNs) [47] with Hi-C at each of these major cell transition stages. We found that the *Ebf3* promoter makes strong contacts across a large region encompassing mouse hs737 (mo-hs737) and that these interactions become stronger during differentiation to NPCs and CNs (Fig. 4B). We used HiCCUPS [48] to call loops at each stage and found that in CNs there is a ~1.3-Mbp loop that brings mo-hs737 into close proximity with the *Ebf3* promoter (loop anchors chr7:136,050,000–136,075,000 and chr7:137,300,000–137,325,000). We did not observe loops between mo-hs737 and any other genes on the chromosome.

Next, analyzing the human corticogenesis data which was generated by dissecting three fetal brain samples into cortical plate (CP) and germinal zone (GZ) layers [49], we created chromatin contact maps using Juicer [48] for each dissection layer and called loops again using HiCCUPS [48]. We find that the *EBF3* promoter interacts with the region of hs737 in both the GZ (loop anchors chr10:128,535,000–128,540,000) and CP (loop anchors chr10:128,525,001–128,550,00 and chr10:129,995,001–129,975,000) samples. We note that there is a loop in CP between hs737 and a second gene *GRLX3* (loop anchors ch10:128,525,001–128,550,000 and chr10:130,125,001–130,150,000), a gene known to be involved in multiple mitochondrial dysfunctions syndrome (Fig. 4A). However, the loop between hs737 and *GLRX3* is both weaker and less significant than the one formed between hs737 and *EBF3*.

Thus, narrowing in on *EBF3*, we searched recent literature on coding DNVs and found *EBF3* is the only gene in this TAD that has known statistical enrichment for coding DNVs in NDDs [50, 51]. Combining data from these two previous studies (Fig. 4C, D; Supplemental Table S8), we also saw genome-wide significance (chimpanzee-human [50] missense p = 8.12 × 10^−35^, chimpanzee-human loss-of-function p = 2.26 × 10^−13^, denovolyzeR [52] missense p = 4.79 × 10^−13^, denovolyzeR loss-of-function p = 7.97 × 10^−22^) for coding DNVs using two different statistical tests. This is also the gene for the Mendelian phenotype hypotonia, ataxia, and delayed development syndrome (HADDS [53]) and has been characterized in detail in a set of ten patients [54]. Searching the GTEx database, we find that *EBF3* is widely expressed throughout the body and in the brain (Fig. 6A) and similarly find in the Human Protein Atlas that EBF3 is detected in many tissues including the brain (Fig. 6B), whereas hs737 displays much more restricted activity only being active throughout the midbrain and hindbrain (Fig. 6C).

### EBF3 regulates many NDD-significant genes

**Fig. 5:**
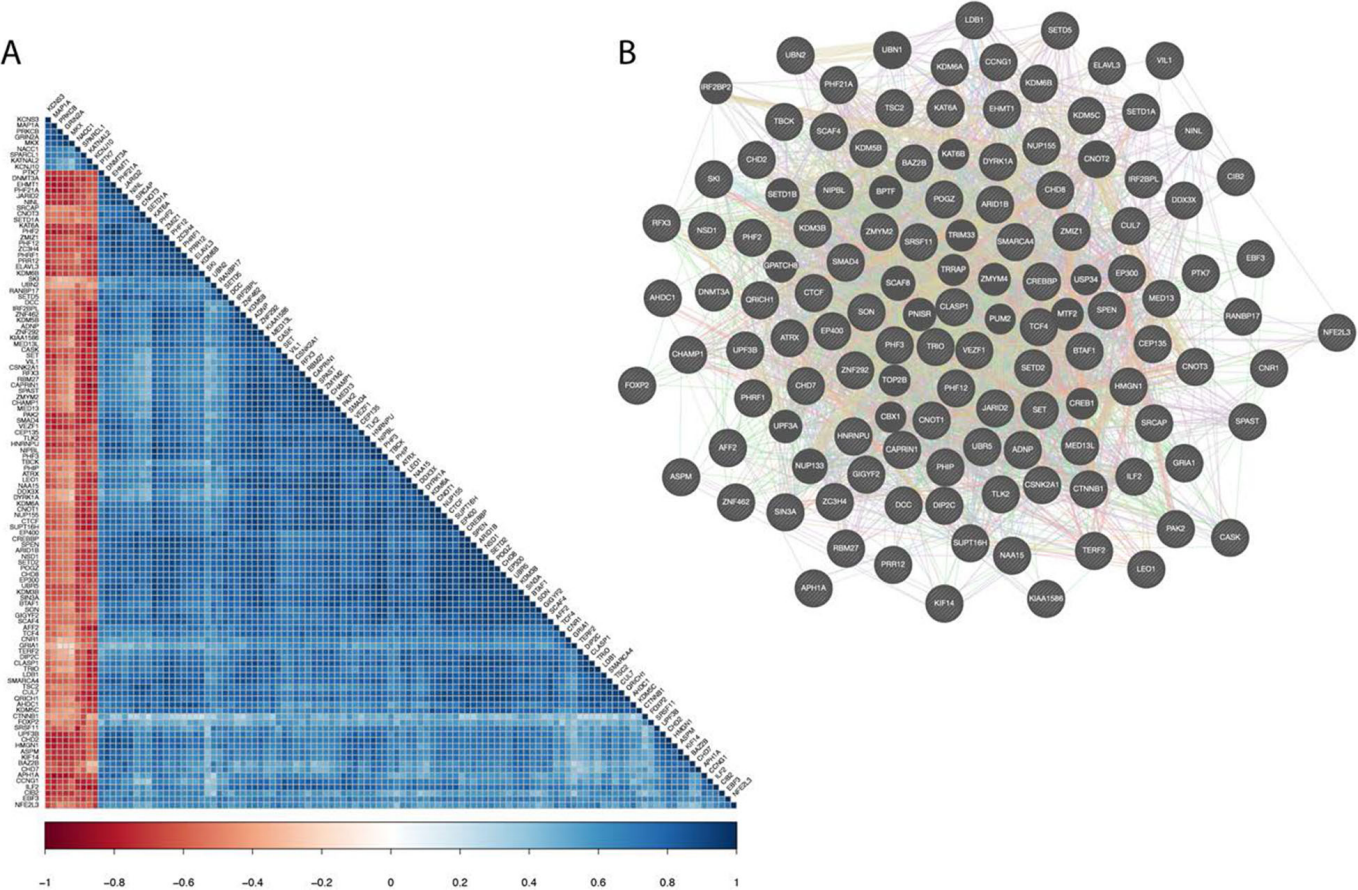
*EBF3* gene network analysis. **A** Correlation matrix of *EBF3* with other high scoring SFARI genes (score < 3) after performing regression and hierarchical clustering and having an absolute correlation greater than 0.6. There is a significant enrichment of genes involved in chromatin binding. **B** Network analysis of genes from cluster 2 from Genemania where each gene is a node and different forms of supporting evidence for an interaction are edges

To assess the global transcriptional control of EBF3, we analyzed its genome-wide bonding profile from chromatin immunoprecipitation sequencing (ChIP-seq) in the human neuroblastoma cell line SK-N-SH [55]. We mapped all EBF3 peaks to promoters to detect EBF3 binding and identified 3100 genes (16% of all genes in the genome) bound by EBF3. We then focused in on genes with statistical enrichment for coding DNVs in NDDs and that were bound by EBF3 (Supplemental Table S9). Of the 253 significant NDD genes in Coe et al. [50], 26.9% of them were bound by EBF3 at their promoter (p = 8.95 × 10^−6^, OR = 1.93). Of the 285 significant genes in another study [51], 26.3% of them were bound by EBF3 (p = 7.43 × 10^−6^, OR = 1.87) (Fig. 4E). Many of these bound NDD genes (Supplemental Table S8,9) are involved in chromatin regulation (Chromatin Binding Gene Ontology p = 3.2 × 10^−7^) and/or transcription factor activity (DNA Binding Gene Ontology p = 6.4 × 10^−11^) (e.g., *CHD8*, *CHD2*, *ARID1B*) indicating that EBF3 may be a master-regulator of many NDD genes. Chromatin binding genes account for a sizeable fraction of DNM attributable cases of autism [12, 14, 56], suggesting that *EBF3* disruption could result in a milder phenotype in the spectrum, as we observe in these cases. We find further support for this by observing that *EBF3* expression is highly correlated (r > |0.6|) with 116 high confidence SFARI genes (SFARI score < 3) (Fig. 5A). Within the cluster of genes positively correlated with *EBF3*, there is a significant enrichment of genes involved in nucleosome organization (FDR p = 3.95 × 10^−2^), regulation of histone modification (FDR p = 2.12 × 10^−2^), chromatin remodeling (FDR p = 2.23 × 10^−2^), chromosome organization (FDR p = 3.00 × 10^−7^), chromatin organization (FDR p = 6.58 × 10^−7^), and positive regulation of the cell cycle process (FDR p = 3.68 × 10^−2^). Performing a network analysis using String-db, we find that the genes positively correlated with *EBF3* have significantly more interactions than expected (PPI enrichment p-value < 1.0 × 10^−16^, expected edges 95, observed edges 202) (Supplemental Figure 3). However, *EBF3* is only connected in this network using a low confidence threshold for connectivity, so we also performed a second network analysis using GeneMania (https://genemania.org) and find *EBF3* is part of the network with evidence from genetic interactions (Fig. 5B).

### Phenotypes of individuals with DNV in hs737

In order to understand more about the phenotypic consequences of variation in the enhancer, we reviewed de-identified phenotype information for each of the individuals with autism that had a DNV in hs737. As gene discovery in simplex autism, based on large CNVs and coding DNVs, has yielded the most findings in females and in individuals who have intellectual disability (full-scale IQ < 70) [12], the first two phenotypes that we assessed were sex and the full-scale IQ scores. All three individuals were male and their full-scale IQs were 103, 89, and 132, respectively (Fig. 1A), suggesting that none of the individuals had an intellectual disability. We also found that all three individuals had evidence of motor problems and/or hypotonia.

**Fig. 6:**
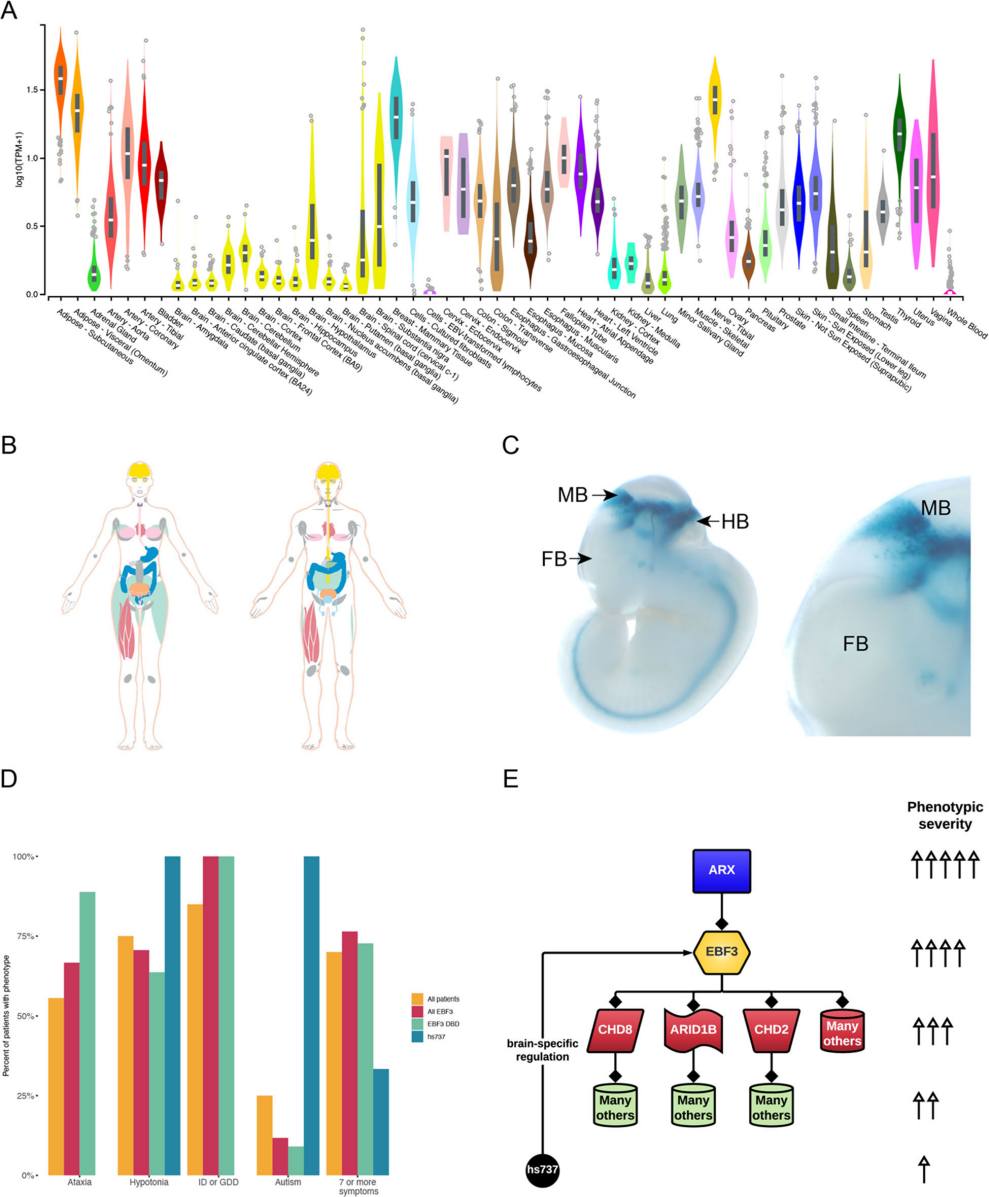
Consequence of coding and noncoding variation in *EBF3*. **A** GTEx expression data of *EBF3* with color corresponding to the organ system. **B** Human Protein Atlas highlighting where *EBF3* expression is detected in the human body. **C** LacZ staining assay for reporter activity driven by the hs737 enhancer at mouse E11.5. **D** Phenotypic analysis comparing the frequency of ataxia, hypotonia, ID and GDD, autism, and having 7 or more symptoms between all patients, individuals with *EBF3* mutations, individuals with mutations specifically in the *EBF3* DNA binding domain, and in the hs737 enhancer. **E** Gene regulatory network encompassing EBF3 built using current molecular biological knowledge

Comparing the individuals with noncoding variants to 14 previously published individuals (13 probands) [54, 55] and 7 new individuals with coding DNVs in *EBF3*, we find that those with coding mutations in *EBF3* typically have more severe phenotypic consequences (Fig. 6D). All individuals with a coding DNV in *EBF3* regardless of position within the protein presented with an intellectual disability or global developmental delay, while no individuals with a DNV in hs737 had either of these phenotypes. Individuals with DNVs in *EBF3* had higher rates of ataxia compared to all individuals with DNVs in *EBF3*, while this phenotype was also absent in individuals with hs737 DNVs. Diagnoses of cerebellar vermis hypoplasia were also found in 4/10 individuals with *EBF3* DNVs who had a brain MRI. Within the *EBF3* mutation group, there are significantly higher rates of intellectual disability or global developmental delay compared to the hs737 group (p = 0.00088) and we find significantly higher rates of autism within the hs737 DNV group when compared to the *EBF3* group (p = 0.0088) (Fig. 6D) (Supplemental Table S10).

## Discussion

Eleven years ago, the Simons Simplex Collection began [57] and started its efforts to understand the role of genomic variation in simplex autism. It was hypothesized that there is a contribution from DNVs in these simplex autism families [57]. Over the 11 years, microarray [18, 20, 58], WES [12–14, 16], and now WGS data [8–10, 24, 25] have been generated to fuel this discovery. The first fruits of these efforts were large copy number variants and coding DNVs. They have turned out to be critical for explaining ~ 30% of individuals with autism [12]. Intriguingly, these variants have been found to be enriched more in females and/or individuals who also had intellectual disability [12]. In particular from this work, coupled with the study of DNVs in neurodevelopmental disorders more broadly, there are now > 100 genes with genome-wide significance for excess of coding variation [50, 51, 59] including the gene *EBF3*. Recent ongoing efforts looking at common variation are providing insights into other aspects of the genetics of autism and explain ~ 50% of autism risk [60]. In our study, we focused on the elusive noncoding DNVs for which we and others have seen aggregate evidence for enrichment in promoters and enhancers [8–10]. We assessed VISTA enhancers in a discovery cohort of 516 families previously published [8] and a replication cohort of 2155 new families. Recent work [11] has indicated the importance of these enhancer regions and we find one reaching nominal significance in the discovery, replication, and combined cohorts (hs737).

We found a hs737 DNV in three different individuals with autism (one in the discovery cohort, two in the replication cohort). The individuals with DNVs had shared phenotypes including being male, intact cognitive function, and all had hypotonia or motor delay (unlike coding DNVs which are enriched for females with intellectual disability [12]). Previous work examining individuals with mutations in the same gene show shared phenotypes [61, 62] much like our individuals with enhancer DNVs. Each DNV had a quantitative effect on reporter gene expression in our in vitro assessment. These DNVs are at highly conserved nucleotides and are predicted to affect binding of transcription factors at the enhancer. Beyond de novo single-nucleotide variants, CNVs encompassing hs737 are also enriched in individuals with NDDs. In our assessment of 28,128 non-NDD individuals, there are none with a deletion or duplication of this enhancer suggesting that it is dosage sensitive in the human population. These lines of genetic evidence support the finding that this enhancer has an important role in the human genome.

Analysis of epigenetic data shows that this enhancer is active in the embryonic brain. A major hurdle in the study of enhancers is determining which gene they regulate. This is especially relevant when enhancers are very distal from the promoter they target. Since hs737 resides in a large noncoding region, we utilized an innovative approach of combining constraint information for genes within the TAD, enrichment of coding DNVs in genes in the TAD, and chromatin contact data. This analysis led to our identification of *EBF3* as the gene targeted by hs737, in both embryonic mouse and human fetal brain, with an interaction across a distance of ~ 1.4 Mbp in the human fetal brain. *EBF3* is a well-established NDD gene with genome-wide significant enrichment of coding DNVs and an established syndrome called HADDS that shares the phenotype of hypotonia with the individuals with autism in our study that had hs737 noncoding DNVs. HADDS is a severe phenotype and affects many parts of the body. This is likely because EBF3 is a transcription factor that is ubiquitously expressed in humans [63] (Fig. 6A, B). It affects many genes in the genome and in particular, in neuronal cells, is enriched for regulating other NDD genes involved in further regulation. We speculate that since hs737 is a brain-specific enhancer that is why we see a less severe phenotype in the individuals with autism in our study than in HADDS (Fig. 6C–E). Aggregating information from the literature and from this current study, we can begin to build the gene regulatory network containing *EBF3* (Fig. 6E) and in this network we can see the importance of genotypic and phenotypic assessment of individuals. In particular, the higher the location of the variant in the network, the more severe the phenotypic consequence (e.g., mutation in ARX), and the lower in the network, the less severe the phenotypic consequence (e.g., mutation in the hs737 enhancer). Along with this observation, we point out the critical work of completing the allelic series for each of the genome-wide significant NDD genes. For *EBF3*, we show that coding and noncoding DNVs result in more and less severe phenotypic outcomes. This type of gene regulatory network building that incorporates coding and noncoding variation is essential for understanding the etiology of autism. For continued delineation of this gene regulatory network, it will be critical to move beyond the in vitro work to in vivo models as in vitro models can be a limitation when studying noncoding elements with specific spatiotemporal activity.

## Conclusions

We identify hs737 as an enhancer with excess DNVs in autism and find through several approaches that it is brain-specific and targets the gene *EBF3*. This study provides critical insights into noncoding DNVs in autism and how they can have similar and differential effects on phenotypic outcomes. This work provides a framework for both future studies of noncoding DNVs and considerations of effects at the level of gene regulatory networks.

## Methods

### DNVs in 2671 autism families

We accessed DNV data from Wilfert et al. [30] through SFARI Base (accession: SFARI_SSC_WGS_2a, https://base.sfari.org/).

### Statistical assessment of DNVs

A list of VISTA enhancers driving the expression of their target genes in the brain was downloaded from the VISTA enhancer browser [27]. DNVs were annotated to each enhancer using bedtools [64]. We modified the fitDNM statistical approach (https://github.com/TNTurnerLab/fitDNM) [29], a method to assess the excess mutational load of DNVs using variant-specific mutation rates calculated based on local sequence context, now applied to noncoding variants in the VISTA brain enhancers.

### Copy number assessment of hs737

To test copy number variant enrichment in morbidity map [35], we downloaded Supplementary Dataset 1 from Coe et al. [35] and identified the window in the genome containing hs737. We report in this paper the case counts, control counts, and p-values for deletions and duplications in this window.

The QuicK-mer2 [37] (https://github.com/KiddLab/QuicK-mer2) workflow was run on WGS data to generate copy number estimates, in 1-kbp windows across the genome, in each individual. Briefly, this method utilizes a kmer-based approach to perform copy number estimation. After running QuicK-mer2, we utilized the bedtools [64] map function to calculate the average copy number across the copy number windows covering hs737 (b38, chr10:128,568,604–128,569,741). If the copy number was less than 1.3, we called it as a deletion and if it was greater than 2.7, we called it as a duplication. QuicK-mer2 was run on the autism families in this study and also the high-coverage 1000 genomes project data available as described at http://ftp.1000genomes.ebi.ac.uk/vol1/ftp/data_collections/1000G_2504_high_coverage/.

To assess structural variation in gnomAD v2.1 [40], we queried for our enhancer region on hg19 (10-130366868-130368005) and also available at this link https://gnomad.broadinstitute.org/region/10-130366868-130368005?dataset=gnomad_sv_r2_1. There were “No variants found” in this region.

### Statistical testing of coding DNVs in *EBF3*

DNV data was collected from two recent papers on NDDs [50, 51]. After overlapping samples between the two studies were removed, there were a total of 37,692 sequenced parent-child trios. To test for enrichment of coding DNVs that were loss-of-function or missense, we applied the chimpanzee-human [50] and denovolyzeR [52] models as previously described [50, 65].

### Mouse ENCODE chromatin state and interaction tracks

Chromatin state and interaction data from mouse developmental timepoints were assessed in the ENCODE Regulation, ENC+EPD Enhc-Gene, ENCODE cCREs, and EPDnew Promoters tracks in the mm10 genome browser at UCSC [42–45].

### ChIP sequencing assessment of data from Harms et al. [55]

We downloaded ChIP sequencing data from Harms et al. [55] at the following GEO link https://www.ncbi.nlm.nih.gov/geo/query/acc.cgi?acc=GSE90682. To identify the promoter locations in the human genome, we looked at sequence 5 kbp upstream of the transcription start site using the Table Browser feature of the UCSC Genome Browser [42]. We then used bedtools [64] intersect to identify which ChIP peaks overlapped with promoters in the human genome. To determine which NDD genes were bound at their promoter, we pulled the genome-wide significant gene lists from Coe et al. [50] and Kaplanis et al. [51] and compared to our EBF3 bound promoter list. Gene Ontology enrichment was performed using the Database for Annotation, Visualization and Integrated Discovery tool version 6.8 (https://david.ncifcrf.gov/) [66].

### RNA sequencing at mouse embryonic day 11.5 from ENCODE

Mouse E11.5 forebrain RNAseq data was downloaded from https://www.encodeproject.org/files/ENCFF465SNB/@@download/ENCFF465SNB.tsv, mouse E11.5 midbrain RNAseq data was downloaded from https://www.encodeproject.org/files/ENCFF359ZOA/@@download/ENCFF359ZOA.tsv, and mouse E11.5 hindbrain data was downloaded from https://www.encodeproject.org/files/ENCFF750FTK/@@download/ENCFF750FTK.tsv. For each file, we retained all Ensembl gene identifiers and annotated them to HGNC-approved identifiers using biomart (https://m.ensembl.org/info/data/biomart/index.html). We used an expression cutoff of > 2 to call a gene as expressed and < 2 as not expressed in each region of the brain.

### Cell lines

Neuro2a (ATCC CCL-131) were purchased from ATCC and grown under standard conditions (DMEM + 10% FBS and 1% penicillin-streptomycin).

### Luciferase assays

Five hundred nanograms of firefly luciferase vector (pGL 4.23, Promega Corporation) containing the enhancer sequence cloned upstream of *luc2* and 9 ng of *Renilla* luciferase vector (transfection control) were transiently transfected into the Neuro2A cell line (1 × 10^5^ cells/well) using 3μl FuGene HD transfection reagent in 100μl of OPTI-MEM medium. Neuro2A cells were incubated for 48 h and luminescence measured using a Dual-Luciferase Reporter Assay System on a Promega GloMax luminometer. All assays were performed in triplicate for a total of six independent readings of each construct. Significance was calculated by using a two-sided t-test assuming unequal variance and used in two situations, first to compare the wild type construct to basal construct and second to compare the variant constructs to the wild type (Supplemental Table S5).

### Transcription factor binding predictions

Transcription factor binding analysis was performed using QBiC-Pred [34] and selecting all transcription factor families and using a p-value threshold of 0.0001 and output to a VCF format. Once predictions were obtained, transcription factors were then cross referenced with RNA sequencing from mouse embryonic brains at day 11.5 to identify which transcription factors are highly expressed.

### Brainspan RNAseq analysis

RNAseq data (RNA-Seq Gencode v10 summarized to gene (n = 52,377 genes)) was downloaded from http://brainspan.org/static/download.html on 4 February 2021 for the developmental transcriptome which contains samples from 8 weeks after conception to 40 years. Linear regression was performed on the data set for age and brain region using R. Spearman correlations were calculated for each gene (X) and *EBF3* (Y) and SFARI genes with an absolute correlation greater than 0.6 were retained for further analysis. Pathway analysis was performed using GeneMania (https://genemania.org) and String-db (https://string-db.org). GOTERM analysis was performed using http://geneontology.org where the input genes were those from cluster positively correlated with *EBF3* and SFARI gene list (1/13/21 release) was used as the background.

### Statistical assessment of phenotypes

Phenotypes were assessed by counting the number of individuals with a given phenotype. Specifically, we assessed all probands from previous studies for a total of 13 probands in addition to 7 new probands. The 7 or more symptoms category was calculated as the total number of neurological abnormalities, other diagnoses, and the presence of craniofacial abnormalities, where 1 was added to the total number of symptoms if the individual had at least one craniofacial abnormality. A minus (−) value was taken to be the absence of the phenotype whereas NA was taken to be that the assessment was missing. Two probands were missing assessments for ataxia and were excluded from that analysis for a total of 18 individuals rather than 20. A one-sided Fisher’s exact test was used to calculate significance and odds ratio for the phenotypes and only probands who had an assessment for that phenotype were included in the calculation.

## Supplementary Information

**Additional file 1: Table S1**: Coordinates in the human genome (build 38) of VISTA enhancers driving expression in the brain.

**Additional file 2: Fig S1**. Polygenic risk scores (PRS) for the three individuals with hs737 mutations. **Fig. S2**. Zoom in on the hs737 enhancer with annotations from other datasets. The enhancer is in a PsychEncode fetal enhancer, contains central nervous system DNaseI hypersensitive sites, contains conserved transcription factor binding sites, and is highly conserved across the vertebrate lineage. Also shown are the locations of the hs737 *de novo* mutations identified in individuals with autism. **Fig S3**. String-db network analysis, predicts interactions between *EBF3* and *SPAST, CSNK2A1, HNRNPU, CHD7, KDM6A, KDM6B.* **Table S2**. fitDNM results for *de novo* mutations in VISTA enhancers driving brain expression. **Table S3**. Other *de novo* SNVs/indels seen in individuals with hs737 enhancer mutations. **Table S4**. Other copy number variation in individuals with hs737 *de novo* mutations. **Table S5**. Statistical significance calculations for the luciferase assays. **Table S6**. Expression of transcription factors potentially binding at variant locations in the hs737 enhancers.

**Additional file 3: Table S7:  **should have the label "CNVs over hs737 in the morbidity map dataset.

**Additional file 4: Table S8**: Protein-coding de novo mutations in EBF3 from Coe et al. 2019, Nature Genetics and Kaplanis et al. 2020, bioRxiv.

**Additional file 5: Table S9: **should have the label "NDD genes bound by EBF3.

**Additional file 6: Table S10: **should have the label "Phenotypes of individuals with coding and noncoding variants affecting EBF3.

## Data Availability

The DNV callset is available at SFARI Base accession: SFARI_SSC_WGS_2a, https://base.sfari.org/. We are grateful to all of the families at the participating SSC sites, as well as the principal investigators (A. Beaudet, R. Bernier, J. Constantino, E. Cook, E. Fombonne, D. Geschwind, R. Goin-Kochel, E. Hanson, D. Grice, A. Klin, D. Ledbetter, C. Lord, C. Martin, D. Martin, R. Maxim, J. Miles, O. Ousley, K. Pelphrey, B. Peterson, J. Piggot, C. Saulnier, M. State, W. Stone, J. Sutcliffe, C. Walsh, Z. Warren, and E. Wijsman). We appreciate obtaining access to phenotypic and genetic data on SFARI Base. Approved researchers can obtain the SSC population dataset described in this study (https://www.sfari.org/resource/simons-simplex-collection/) by applying at https://base.sfari.org.
