# Supplementary material for: Coding and noncoding variants in EBF3 are involved in HADDS and simplex autism
Source: Hum Genomics. 2021 Jul 13;15:44. doi: 10.1186/s40246-021-00342-3 (PMC8278787; doi:10.1186/s40246-021-00342-3)
Supplement: Supplementary file 2 — Additional file 2: Fig S1. Polygenic risk scores (PRS) for the three individuals with hs737 mutations. Fig. S2. Zoom in on the hs737 enhancer with annotations from other datasets. The enhancer is in a PsychEncode fetal enhancer, contains central nervous system DNaseI hypersensitive sites, contains conserved transcription factor binding sites, and is highly conserved across the vertebrate lineage. Also shown are the locations of the hs737 de novo mutations identified in individuals with autism. Fig S3. String-db network analysis, predicts interactions between EBF3 and SPAST, CSNK2A1, HNRNPU, CHD7, KDM6A, KDM6B. Table S2. fitDNM results for de novo mutations in VISTA enhancers driving brain expression. Table S3. Other de novo SNVs/indels seen in individuals with hs737 enhancer mutations. Table S4. Other copy number variation in individuals with hs737 de novo mutations. Table S5. Statistical significance calculations for the luciferase assays. Table S6. Expression of transcription factors potentially binding at variant locations in the hs737 enhancers. [file 40246_2021_342_MOESM2_ESM.pdf]

Fig S1

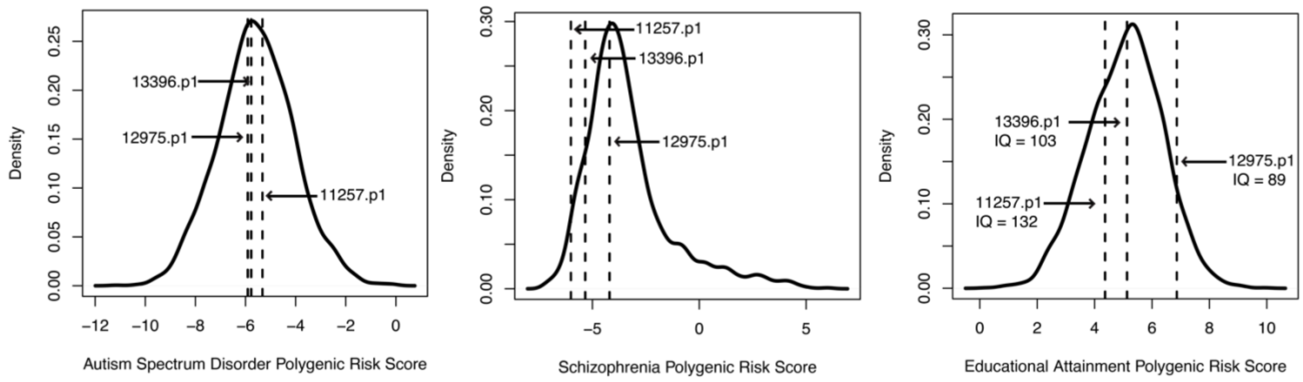

Polygenic risk scores (PRS) for the three individuals with hs737 mutations

Fig. S2.

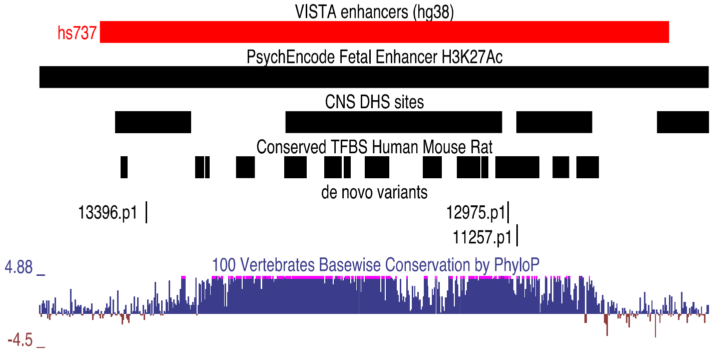

Zoom in on the hs737 enhancer with annotations from other datasets. The enhancer is in a PsychEncode fetal enhancer, contains central nervous system DNaseI hypersensitive sites, contains conserved transcription factor binding sites, and is highly conserved across the vertebrate lineage. Also shown are the locations of the hs737 *de novo* mutations identified in individuals with autism.

Fig S3.

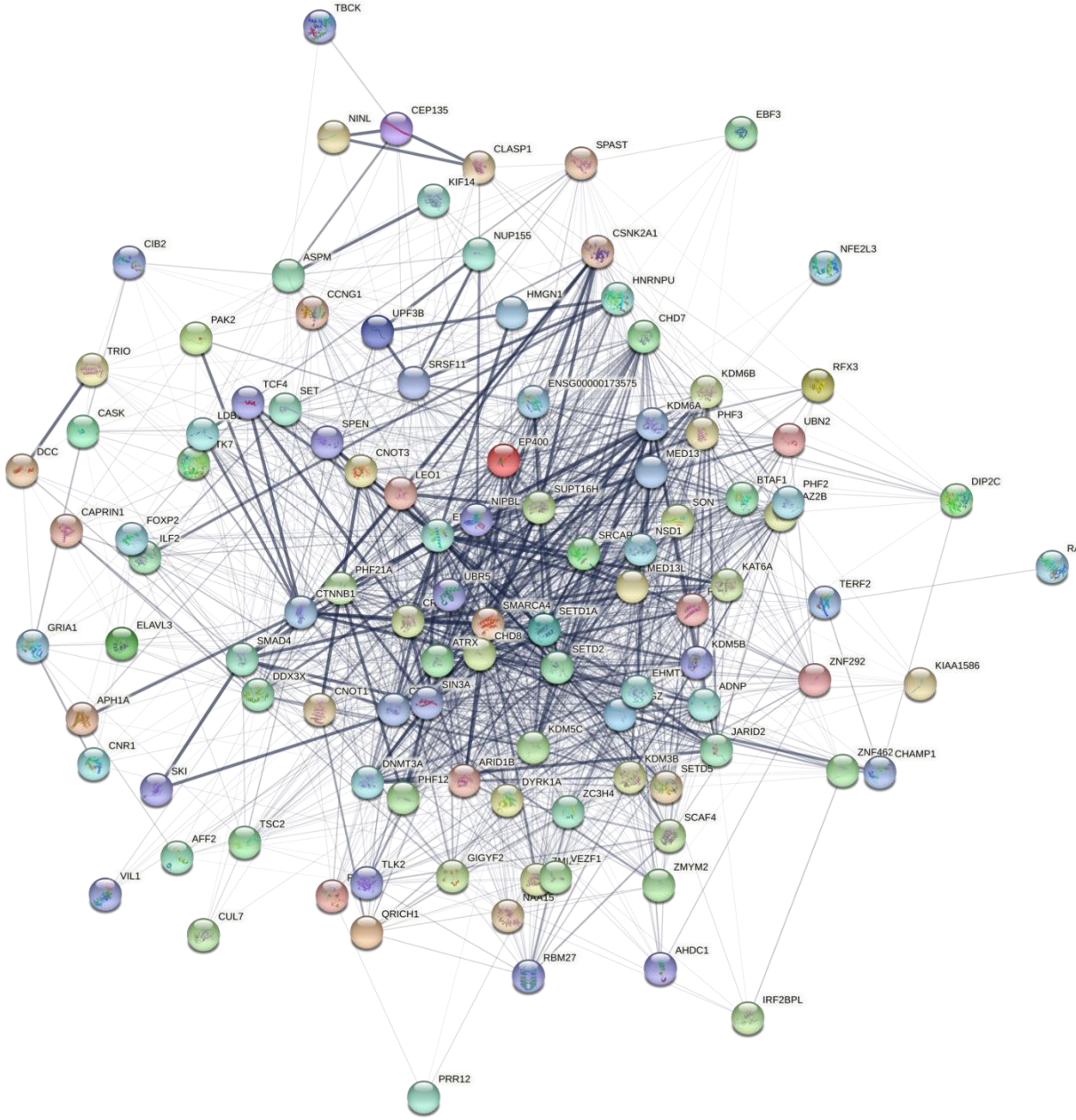

String-db network analysis, predicts interactions between *EBF3* and *SPAST*, *CSNK2A1*, *HNRNPU*, *CHD7*, *KDM6A*, *KDM6B*

Table S2.

enhancerID	nsample.adj	nsnv	nsnv.analysis	ndenovo	score	pvalue.Poisson
hs737	2671	1137	1137	3	1.641	0.00011221
hs2333	2671	2281	2281	3	1.082	0.0003967
hs1330	2671	2148	2148	2	1.226	0.0080814
hs1574	2671	2590	2590	2	1.273	0.01424403
hs1391	2671	2860	2860	2	0.535	0.0171281
hs2543	2671	3181	3181	2	1.294	0.02933883
hs2094	2671	4135	4135	2	0.85	0.0452304
hs357	2671	714	714	1	0.399	0.04595281
hs1	2671	639	639	1	0.43	0.04781171
hs71	2671	858	858	1	0.516	0.06021351
hs816	2671	1113	1113	1	0.173	0.06101038
hs762	2671	901	901	1	0.257	0.06406557
hs967	2671	1177	1177	1	0.334	0.06628823
hs798	2671	1234	1234	1	0.396	0.06913811
hs701	2671	1118	1118	1	0.326	0.07495069
hs573	2671	1308	1308	1	0.162	0.07588227
hs722	2671	1350	1350	1	0.196	0.07789904
hs26	2671	1098	1098	1	0.992	0.07911685
hs759	2671	1329	1329	1	0.485	0.07932293
hs658	2671	1384	1384	1	0.346	0.08087798
hs742	2671	1630	1630	1	0.527	0.09246516
hs312	2671	1367	1367	1	0.41	0.09950883
hs796	2671	1589	1589	1	0.174	0.10073273
hs204	2671	1560	1560	1	0.207	0.11156034
hs2063	2671	1852	1852	1	0.299	0.11468139
hs2563	2671	2743	2743	1	0.231	0.17260254
hs2199	2671	3680	3680	1	0.466	0.25566277
hs2240	2671	4457	4457	1	0.775	0.30321029
hs2207	2671	5388	5388	1	0.654	0.36778757

fitDNM results for *de novo* mutations in VISTA enhancers driving brain expression

Table S3.

VISTA enhancer this individual also has a mutation in	Chromosome	Position hg38	Reference	Alternate	Sample	Annotation	Gene Name	Franklin Input (hg19)	Franklin Classification
hs737	chr7	65954087	C	T	11257.p1	missense_variant	*VKORC1L1*	chr7:65419074-C-T	VUS
hs737	chr3	128087731	A	T	11257.p1	missense_variant	*RUVBL1*	chr3:127806574-A-T	VUS
hs737	chr9	127508511	C	T	12975.p1	missense_variant	*FAM129B*	chr9:130270790-C-T	VUS
hs737	chr20	41416790	C	T	12975.p1	missense_variant	*CHD6*	chr20:40045430-C-T	VUS
hs737	chr11	17771983	G	A	12975.p1	missense_variant	*KCNC1*	chr11:17793530-G-A	VUS

Other *de novo* SNVs/indels seen in individuals with hs737 enhancer mutations

Table S4.

VISTA enhancer this individual also has a mutation in	Franklin input (on hg19)	Franklin_Classification	PatientID	Band	Gender
hs737	DEL:chr11:121264079-122897730	Uncertain	12975.p1	11q24.1	Male

Other copy number variation in individuals with hs737 *de novo* mutations.

Table S5.

Construct	Fold change from basal	Standard error	t-stat	p value
hs737 WT	1.5	1979.3E-4	-2.1389391	0.06488352
hs737.1	5291.0E-4	990.8E-4	4.35923292	0.00331729
hs737.2	3490.1E-4	490.6E-4	5.61490204	0.00136208
hs737.3	4522.9E-4	587.9E-4	5.04514053	0.00234488

Statistical significance calculations for the luciferase assays.

Table S6.

transcription factor	forebrain expression	midbrain expression	hindbrain expression
Sox10	no	no	yes
Sox14	no	yes	yes
Sox8	yes	no	yes
Sox9	yes	yes	yes
Sox17	yes	yes	yes
Sox5	yes	yes	yes
Sox3	yes	yes	yes
Sox18	yes	yes	yes
Sox12	yes	yes	yes
Sox6	yes	yes	yes
Sox21	yes	yes	yes
Sox7	yes	yes	yes
Sox11	yes	yes	yes
Sox13	yes	yes	yes
Sox2	yes	yes	yes
Sox4	yes	yes	yes
Sox1	yes	yes	yes
Arid3b	yes	yes	yes
Arid1a	yes	yes	yes
Arid3a	yes	yes	yes
Arid5b	yes	yes	yes
Arid4a	yes	yes	yes
Arx	yes	yes	no

Expression of transcription factors potentially binding at variant locations in the hs737 enhancers.
